# Supplementary material for: Machine learning-based investigation of regulated cell death for predicting prognosis and immunotherapy response in glioma patients
Source: Sci Rep. 2024 Feb 20;14:4173. doi: 10.1038/s41598-024-54643-3 (PMC10879095; doi:10.1038/s41598-024-54643-3)
Supplement: Supplementary file 1 — Supplementary Figures. [file 41598_2024_54643_MOESM1_ESM.docx]

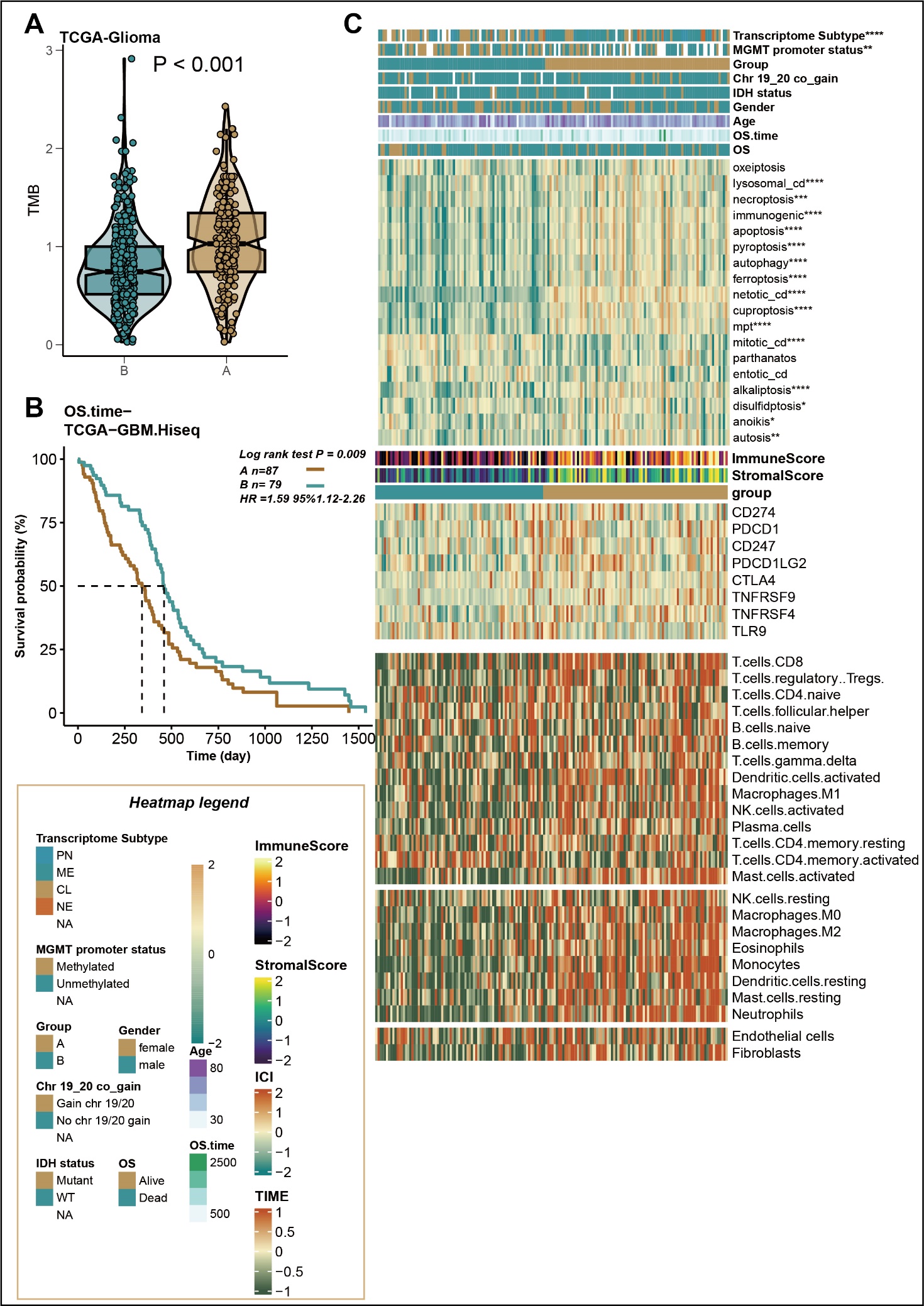


Supplementary fig 1. A. The difference of the tumor mutation burden between the RCD cluster A and RCD cluster B in TCGA-Glioma cohort (TCGA-LGG GBM). B. The K-M curves of the different overall survival for the two distinct RCD clusters in TCGA-GBM cohort. C. A heat map showing the relationship of the RCD clusters and the clinical index, RCD signatures, expression of immune checkpoint genes, and infiltration of the immune cells in TCGA-GBM cohort.


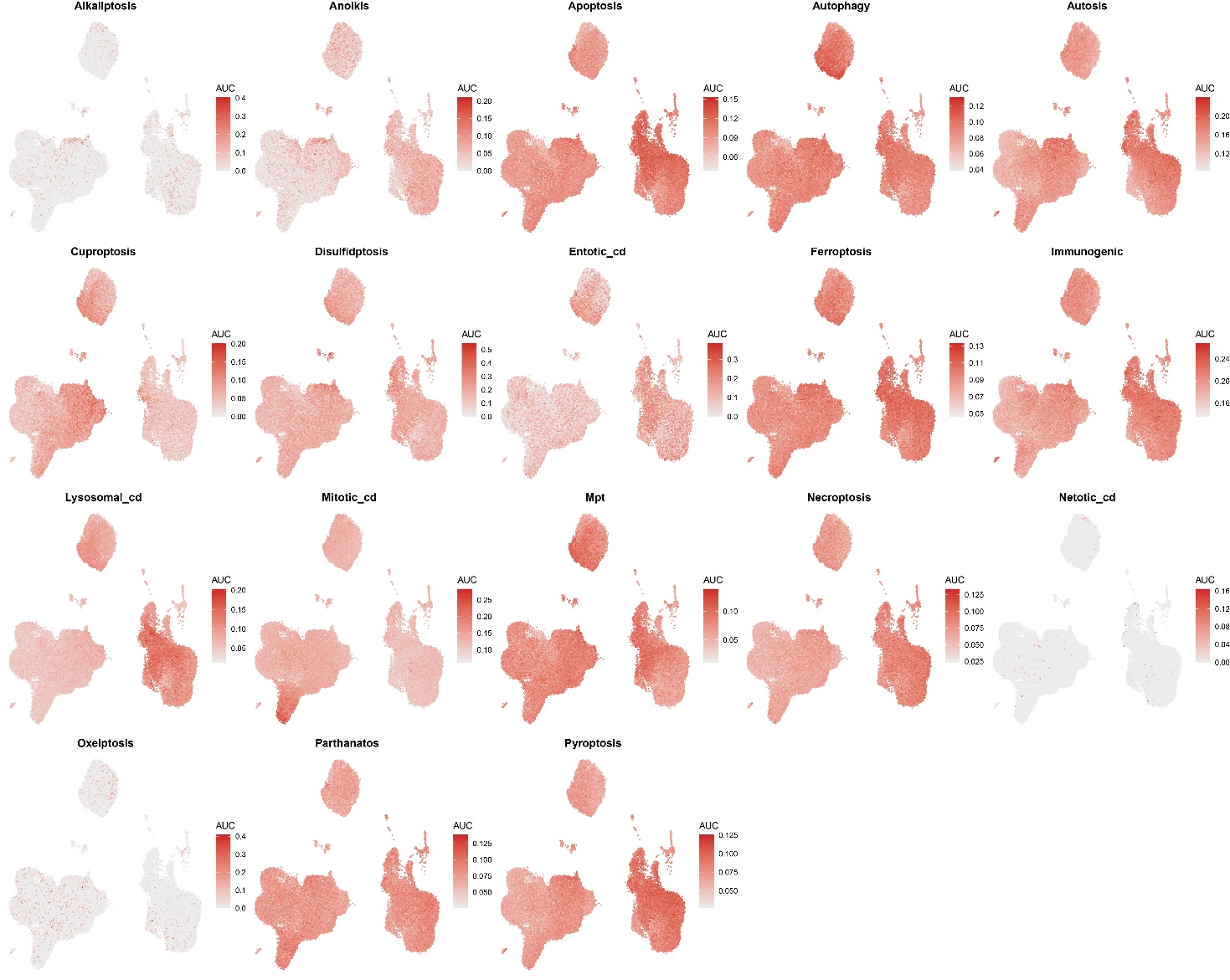


Supplementary fig 2. UMAP views of the AUCell scores of the different RCDs in 50000+ cells.


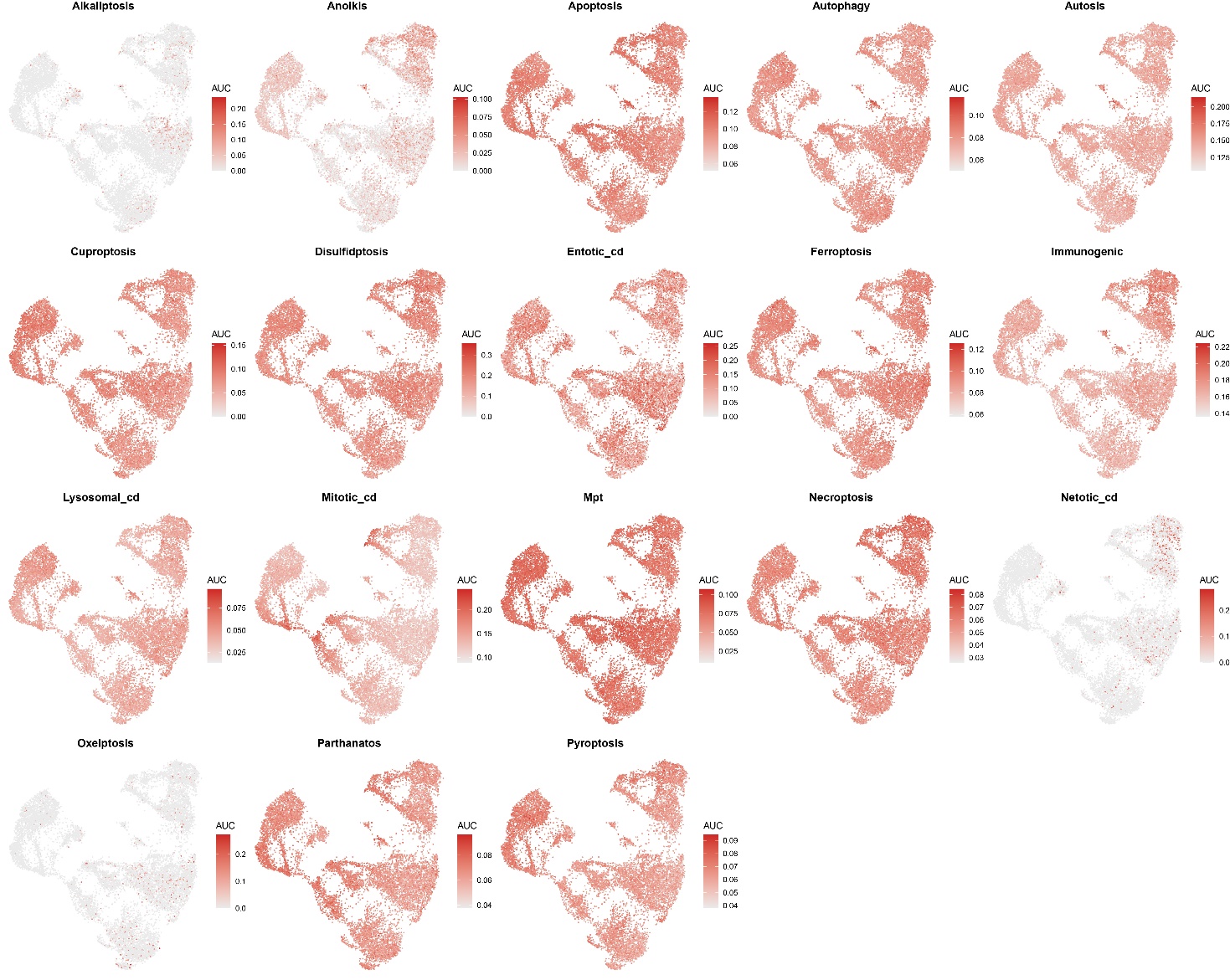


Supplementary fig 3. UMAP views of the AUCell scores of the different RCDs in 10000+ cells which were treated with hypoxia and radiation.


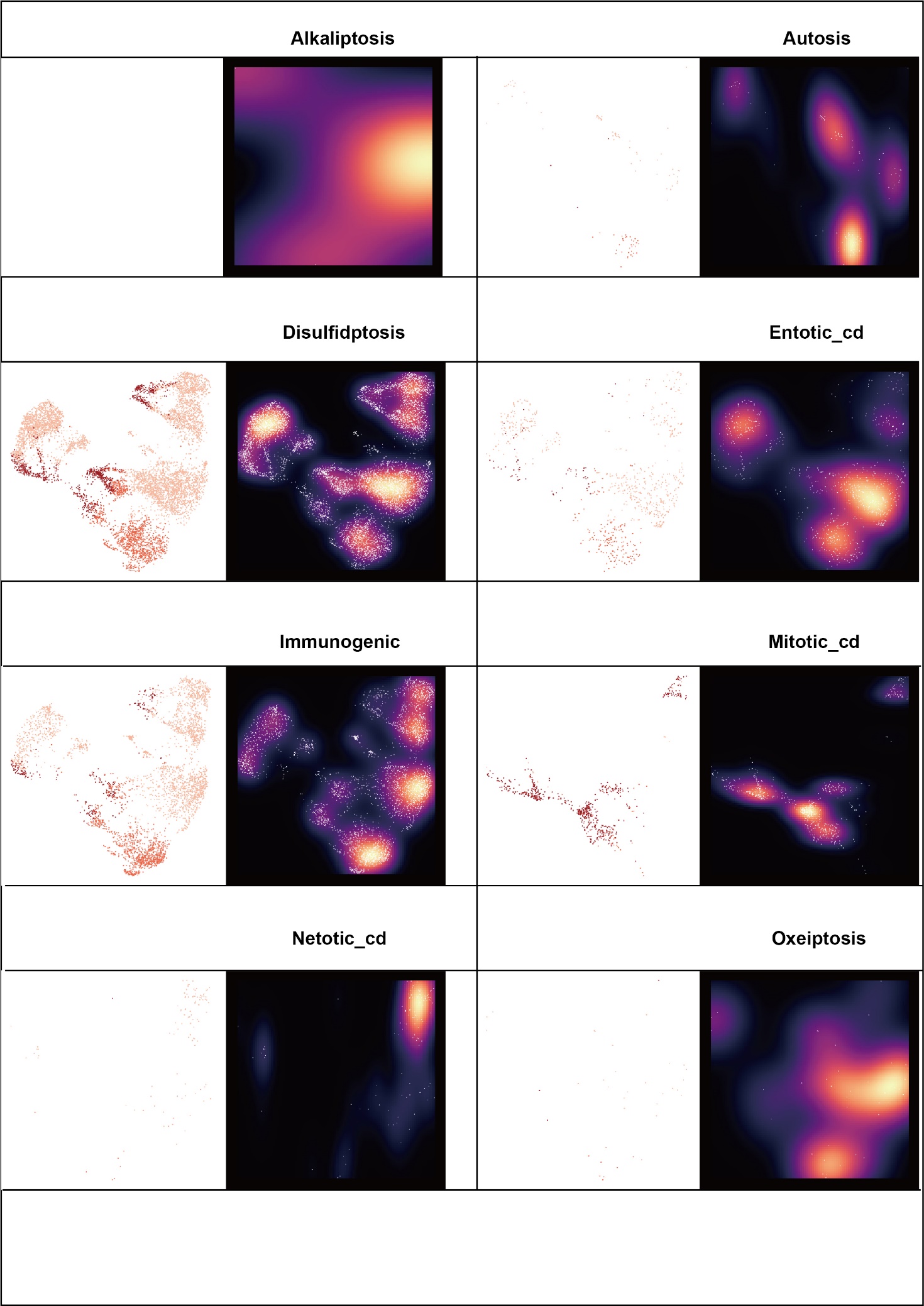


Supplementary fig 4. The distribution and the density of the dominant RCD types in 10000+ cells which were treated with hypoxia and radiation.


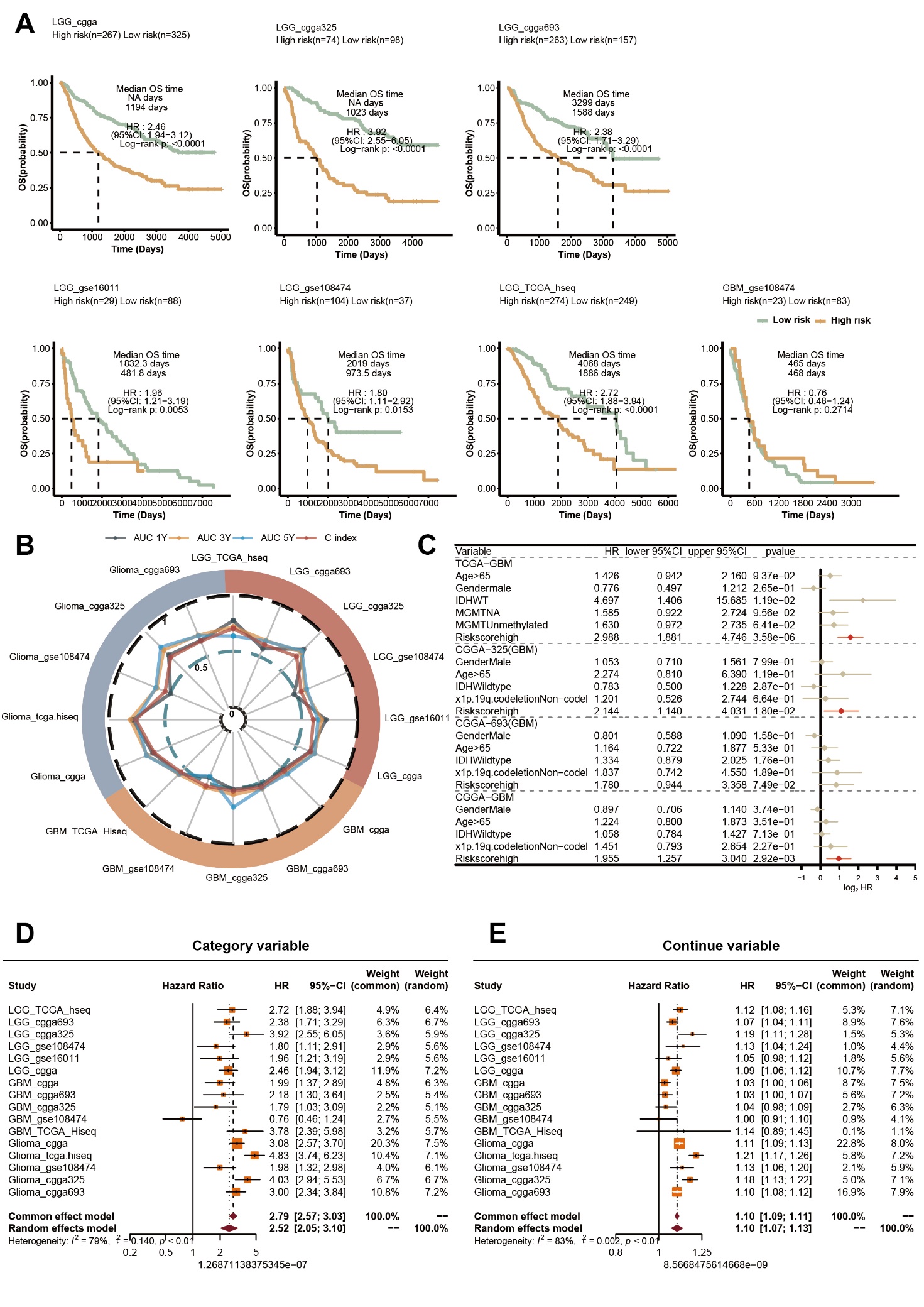


Supplementary fig 5. A. The validation of the RCD.GP scoring system in external glioma datasets. B. The 1-year AUC, 3-year AUC, 5- year AUC and C-index of the RCD.GP score in multiple glioma cohorts. C. Multivariate Cox regression analysis of the RCD.GP score was performed in TCGA-GBM, CGGA-325 GBM and CGGA-693 GBM and CGGA GBM cohort. D-E. The meta analysis of the RCD.GP score for the category (D) and continue (E) variable.


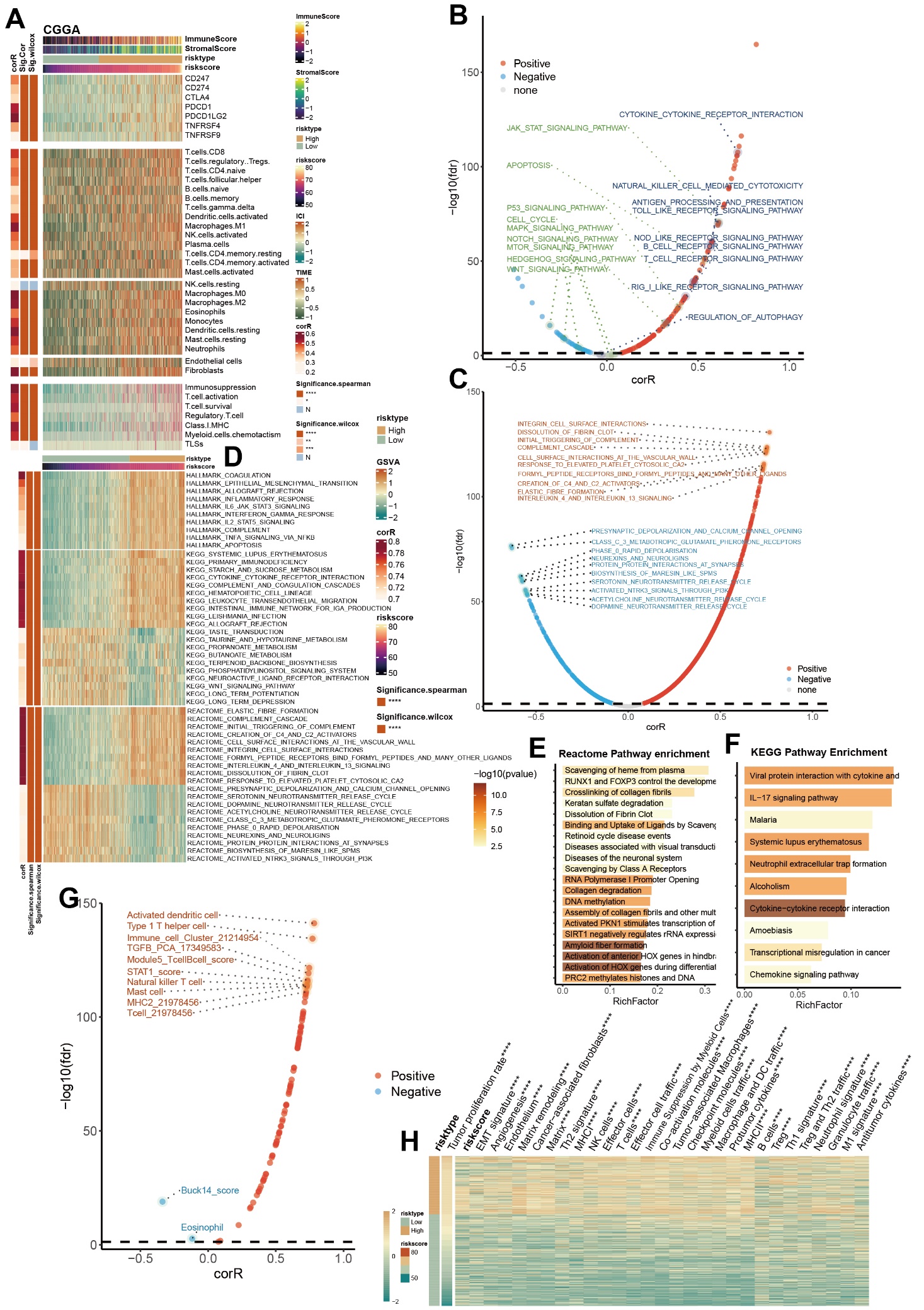


Supplementary fig 6. A. A heat map showing the relationship of the RCD.GP score and the immune score, stromal score, expression of the immune checkpoint genes, infiltration of immune cells and immune microenvironment function in CGGA cohort. B. The spearman’s correlation of the RCD.GP score and pathways associated with proliferation and the immunity in KEGG database. C. The spearman’s correlation between the RCD.GP score and the pathway signal scores in REACTOME database. D. A heat map showing the scaled values of the GSVA scores of the pathways in KEGG, REACTOME, HALLMARK and GO BP database. E. Enrichment analysis of the DEGs between the high risk RCD.GP score subgroup and low risk RCD.GP score subgroup with REACTOME and KEGG pathway database. G. The spearman’s correlation of the RCD.GP score and the immune score related with the immunotherapy response. H. A heat map showing the scaled GSVA scores of the 29 signatures related with tumor microenvironment. The Wilcoxon rank sum test was performed. “*”, “**”, “***”, and “****” represented that the p value < 0.05, 0.01, 0.001, and 0.0001.


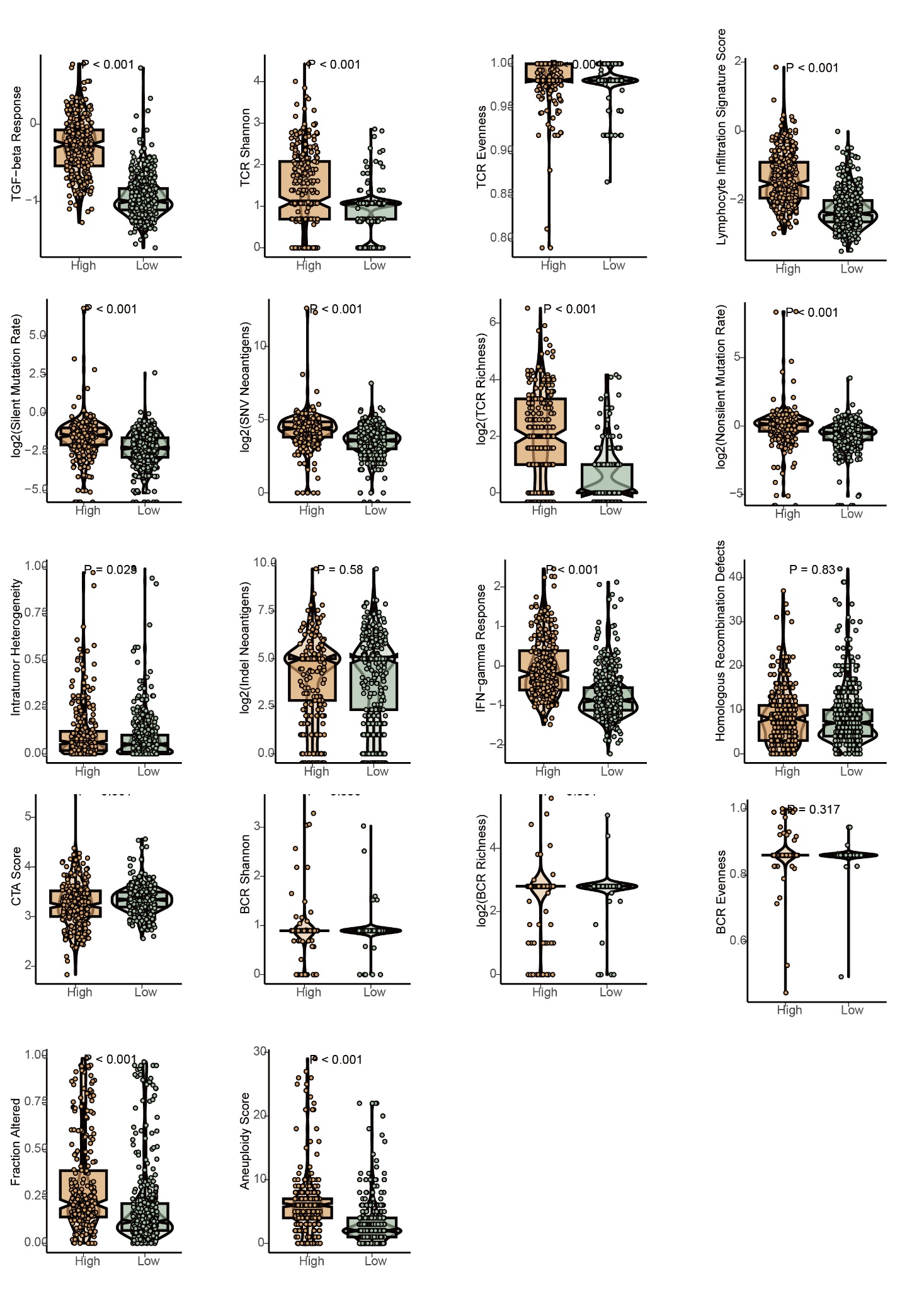


Supplementary fig 7. The difference of the immune scores related with immunotherapy response between the high and low RCD.GP score subgroups. The Wilcoxon rank sum test was utilized.


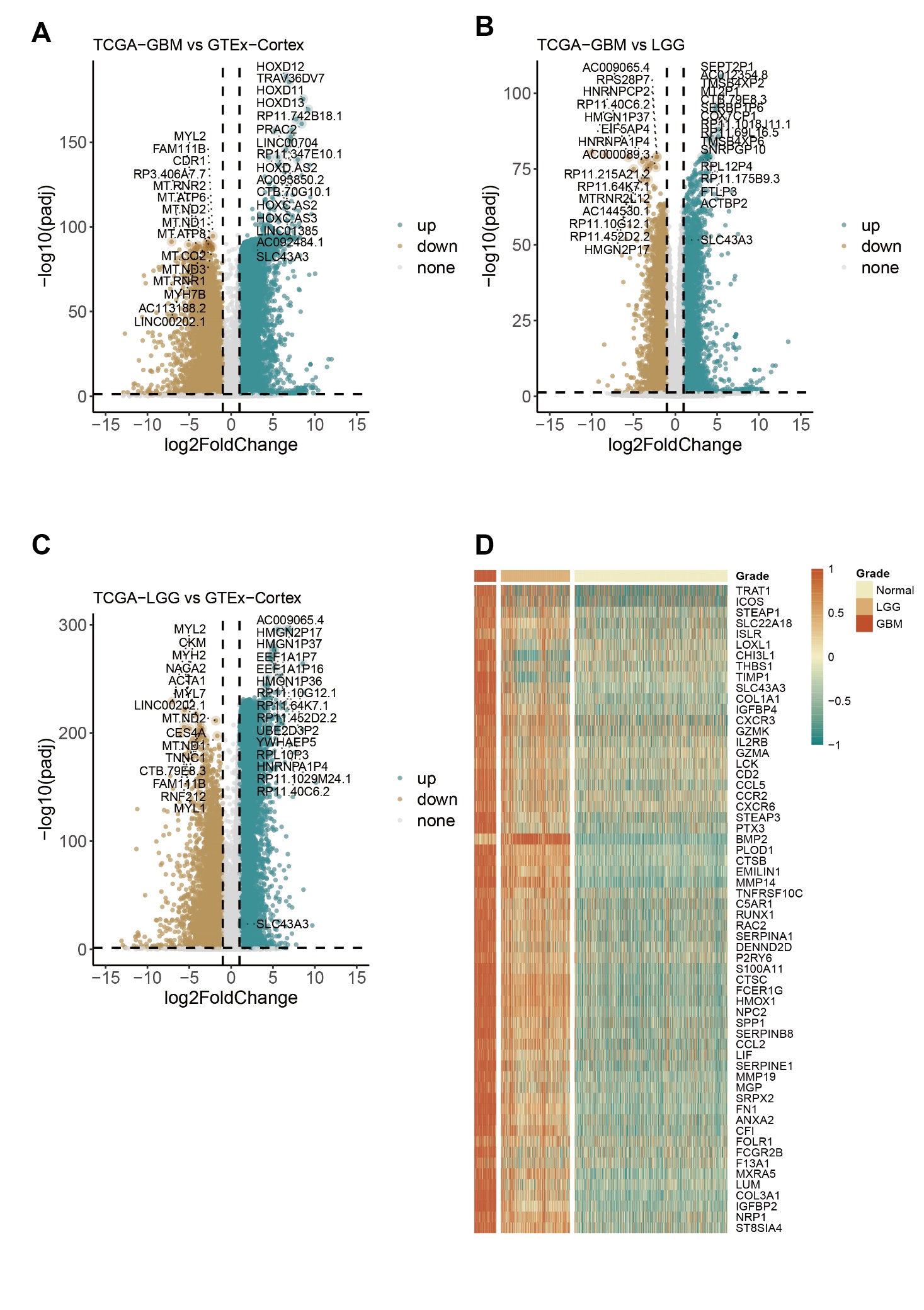


Supplementary fig 8. A-C. The volcano plots showing the DEGs among the GBM, LGG, and normal brain cortex tissues with the TCGA-GBM, TCGA-LGG and GTEx cohort. D. A heat map showing the expression of the 60 prognostic RCD-related DEGs in TCGA-Glioma and GTEx cohort.


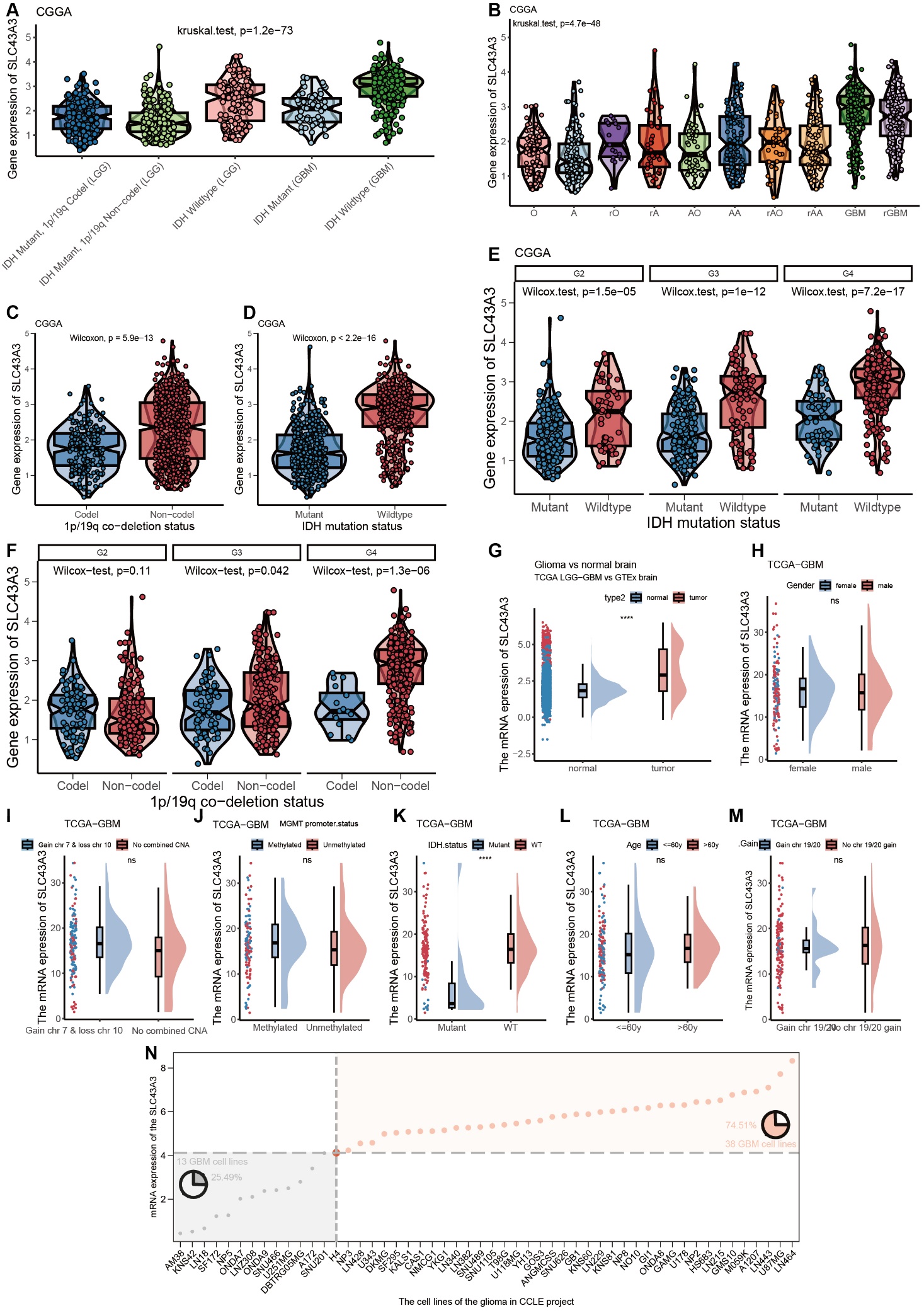


Supplementary fig 9. A-F. The expression of the SLC43A3 in different subtypes of the CGGA cohort. G-M. The expression of the SLC43A3 in different subtypes of the TCGA-GBM cohort. N. The expression of the SLC43A3 in different glioma cell lines. In 74.51% GBM cell lines, the expression of SLC43A3 was higher than in LGG cell line (H4), while in 25.49% GBM cell lines, it was opposite.


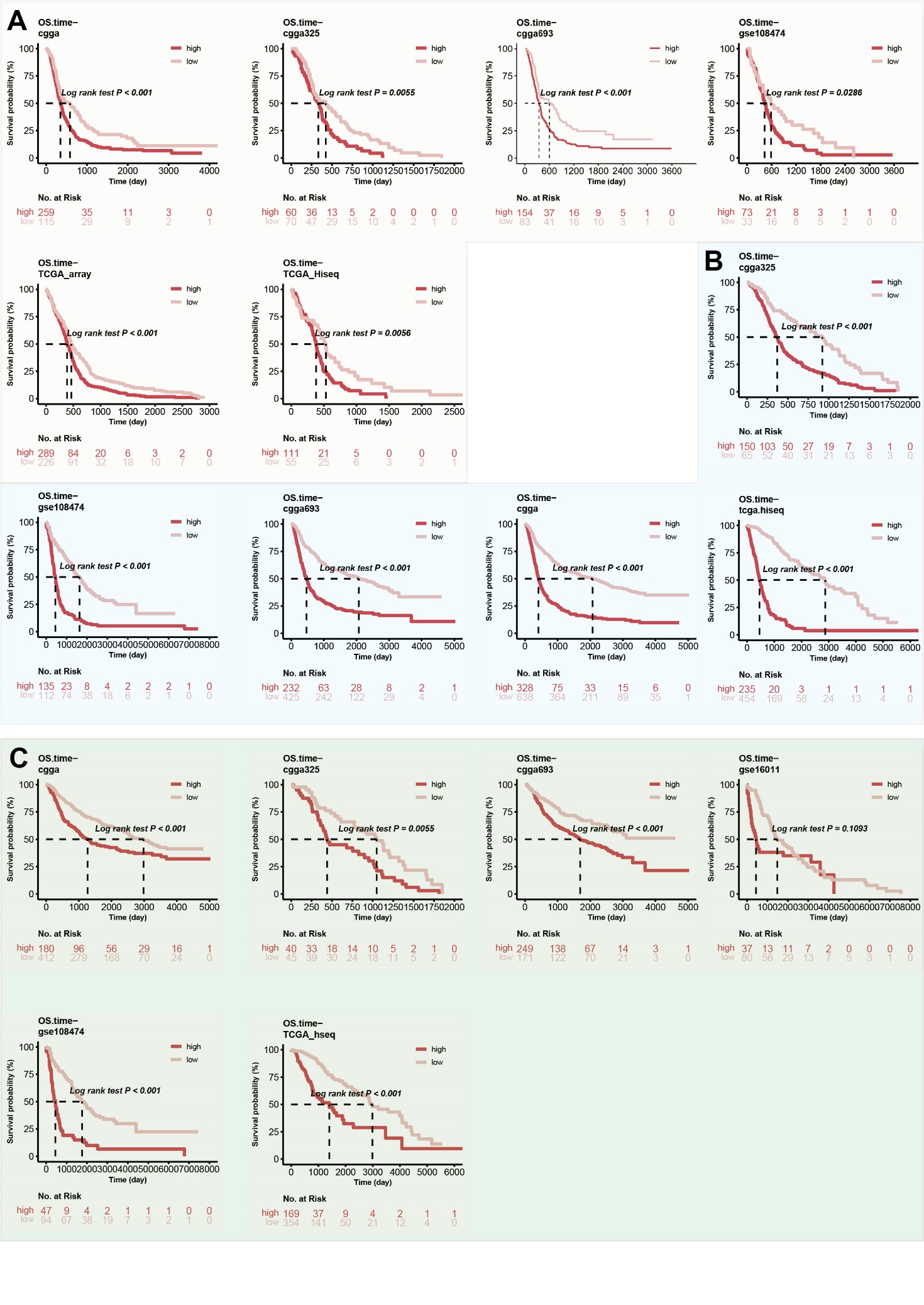


Supplementary fig 10. The K-M curves showing the prognostic values of the SLC43A3 in GBM (A), glioma (B) and LGG (C).


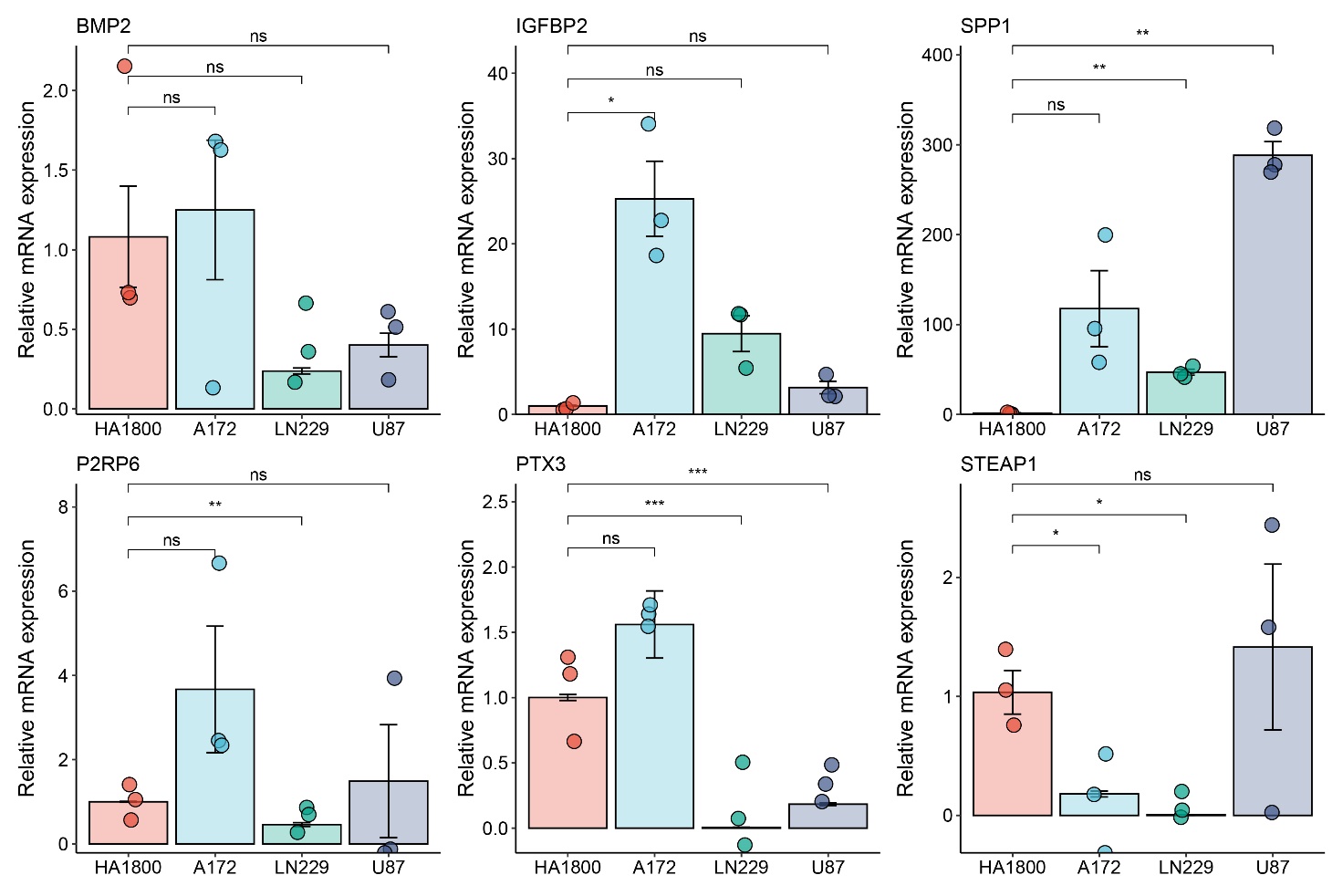


Supplementary fig 11. The qRT-PCR of the expression of screened core genes including BMP2, IGFBP2, SPP1, P2RY6, PTX3, and STEAP1 in different glioma cell lines. The human astrocytes (HA1800) and three GBM cell lines including A172, LN229 and U87 were used for evaluating the expression of the score genes. The t test was utilized for confirming the difference.
